# Engineered Sustainable Mxene-PVA Hydrogel as an Inspiring Co-Delivery Carrier for Targeting Solid Tumors

**DOI:** 10.3390/pharmaceutics17070823

**Published:** 2025-06-25

**Authors:** Elham Ghazizadeh, Mahya Sadeghi, Hans-Peter Deigner, Ali Neshastehriz

**Affiliations:** 1 Department of Bioinspired Materials and Biosensor Technologies, Institute of Materials Science, Faculty of Engineering, Kiel University, 24143 Kiel, Germany; 2 Radiation Biology Research Center, Iran University of Medical Sciences (IUMS), Tehran 1416634793, Iran; 3 Department of Tissue Engineering, Faculty of Advanced Technologies, Tehran University of Medical Sciences, Tehran 14155-6559, Iran; mahya23@gmail.com; 4 Institute of Precision Medicine, Furtwangen University, Jakob-Kienzle-Strasse 17, 78054 Villingen-Schwenningen, Germany; hans78@gmail.com

**Keywords:** bio-inspired hydrogel, drug delivery, solid tumors, miR, Adriamycin

## Abstract

**Background:** Solid tumors have long presented a significant challenge in the field of oncology due to their ability to develop resistance to multiple drugs, known as multidrug resistance (MDR). This phenomenon often leads to treatment failure and poor patient outcomes. In recent years, researchers have been exploring innovative approaches to combat MDR, including the use of hydrogels for localized drug delivery. **Methods:** Through the biological crosslinking of an MB-smDNA-MB agent to form a pH sensitive hydrogel matrix, we introduce the injection coating of a novel PVA-MB-smDNA-MB-Mxene (PMSDMM) carrier for Adriamycin (a potent chemotherapy drug) and miR-375 (as tumor-suppressive microRNA) delivery. **Results:** We aimed to enhance the effectiveness of drug delivery to solid tumors while minimizing systemic toxicity via the pH-sensitive characteristics of methylene blue at the end of smDNA as a dsDNA biological crosslinking agent, i.e., ^anti-miR-375^ PMSDMM _ADR_. Our hydrogel was shown to improve the release of the drug in the acid tumor environment. In the first 24 h, the cumulative release rate was higher at pH = 5.5 than at pH = 7.4. **Conclusions:** We show that this DNA bio-inspired PMSDMM hydrogel has potential in hydrogel injection applications for tumor suppression and tissue regeneration after the surgical resection of tumors.

## 1. Introduction

Solid tumors are a type of cancer that develops from abnormal growths of cells in a specific area of the body, forming a mass or lump. These tumors can occur in various organs such as the breast, lung, colon, or prostate [1]. One of the major challenges in treating solid tumors is multi-drug resistance (MDR), where the cancer cells become resistant to multiple types of chemotherapy drugs. This phenomenon significantly reduces the effectiveness of cancer treatment and poses a serious obstacle to achieving successful outcomes [2,3]. Over time, these cells may develop mechanisms to pump out the drugs, reduce drug uptake, or increase their ability to repair damage caused by the treatment. This leads to a decrease in the concentration of the drugs in the tumor cells, making them less susceptible to the effects of chemotherapy [4]. Addressing multi-drug resistance in solid tumors requires a multi-faceted approach. Researchers are exploring new treatment strategies, such as combination therapies that target multiple pathways involved in drug resistance [5,6]. Additionally, efforts are being made to develop targeted therapies that specifically inhibit the mechanisms responsible for resistance in cancer cells.

Combination therapy is a well-established approach in cancer chemotherapy, and it can also be effective in overcoming MDR [7]. By combining multiple drugs with different mechanisms of action, researchers can target numerous molecular pathways involved in drug resistance simultaneously. This approach disrupts the intricate network of resistance mechanisms, making it harder for cancer cells to develop MDR [8,9]. The co-delivery of microRNA (miR) and one or more drugs refers to the simultaneous administration of two or more therapeutic agents to achieve a synergistic effect [10,11]. Adriamycin, also known as doxorubicin, is a widely used chemotherapy drug for the treatment of various solid tumors. However, prolonged administration of Adriamycin can lead to multidrug resistance, i.e., where cancer cells become resistant to the drug’s cytotoxic effects [12,13]. This phenomenon is often associated with the overexpression of certain genes, including miR-375 [14,15]. Elevated levels of miR-375 have been associated with tumor progression and resistance to chemotherapy. Therefore, targeting miR-375 could be a promising strategy to overcome drug resistance in cancer treatment [16]. Li et al. showed that biodegradable nanoparticles can deliver brinzolamide and miR-375 together as a strategy for glaucoma therapy. This represented a new strategy, with miRNA-124 embedded in PEG-PSA-BRZ nanoparticles. This system showed high encapsulation efficiency (EE), good drug load capacity (DC), and stable control release efficiency (EC) [17]. Men et al. showed that the co-supply of a drug/siRNA combination with mesoporous silicon nanoparticles overcame drug resistance in breast cancer, because the level of expression is increased there. Their study demonstrated the successful delivery of stable-binding Dox and Pgp siRNA to the tumor site [18]. Another study by Liang and colleagues showed that 5-FU and miR-375i, developed on the basis of exosomes, acted as a novel co-delivery system that effectively facilitated cell absorption by significantly reducing the expression of miR-375 in 5-FU-resistant HCT-1165FR cell lines. On the other hand, there are several challenges that need to be overcome concerning the delivery system, notably, that it should have the abilities to protect therapeutic materials from degradation, facilitate cellular absorption, and release the materials at the desired site of action in order to achieve the desired therapeutic effect without causing unwanted side effects [19].

Among the various types of carriers, hydrogel stands out as a distinctive option due to its three-dimensional network of hydrophilic polymers. It has attracted interest in the biomedicine sector because of its unique characteristics [20,21]. Its high water content and soft and flexible nature make it an ideal medium for delivering drugs to specific sites in the body. This characteristic is particularly advantageous when it comes to treating tumors, especially those with MDR [22]. Additionally, hydrogels can protect therapeutic drugs from being degraded by enzymes in the body, thereby enhancing their effectiveness [23]. The potential of hydrogels to overcome tumor MDR lies in their ability to encapsulate chemotherapeutic drugs [24,25]. By encapsulating these drugs, hydrogels protect them from being recognized and eliminated by resistant cancer cells [21]. This allows the drugs to reach their target site and exert their cytotoxic effects. Moreover, the sustained release of drugs from hydrogels prolongs their presence at the tumor site, providing a continuous supply of medication and increasing the chances of eliminating resistant cancer cells [26]. Furthermore, hydrogels can be functionalized to enhance their drug delivery capabilities [27]. For instance, specific targeting ligands can be incorporated into a hydrogel network to facilitate selective drug delivery to cancer cells. Lei and colleagues demonstrated the creation of a hydrogel consisting of a polymeric framework, specifically, ethylene glycol-b-poly (lactic-co-glycolic acid)-b-poly(N-isopropylacrylamide) (PEG-PLGA-PNIPAM). This hydrogel features thermo-responsive mesoporous silicon nanoparticles (MSNs) embedded within a core-shell configuration, aimed at achieving localized and sustained co-delivery of microRNA-222 [28].

Here, we introduce the bio-inspired PVA-MB-smDNA- MB-MXene (PMSDMM) as a hydrogel injection for the co-delivery of Adriamycin and miR-375 to solid tumor sites. This targeted delivery allows for the sustained release of anti-cancer drugs, overcoming the limitations of traditional chemotherapy. PVA’s unique properties, including its biodegradability, mechanical strength, and tunable drug release behavior, make it an attractive choice for effective and controlled drug administration [29]. MXene is part of a recently discovered class of two-dimensional nanomaterials that exhibits exceptional properties, including high surface area with multifunctional groups and remarkable mechanical strength [30]. These properties make MXene an ideal candidate for drug delivery systems [31]. There are many reports on the adsorption of MB in hydrogel networks on the surface of polymer for the removal of MB [32]. Mohammadzadeh et al. showed the effect of the pH sensitive adsorption of MB in a starch-based hydrogel [33]. In our pervious evaluation, we investigated the impact of the biological cross linking of single stranded DNA with hydroxyl and methylene blue ends on hydrogel formation. During this study, we observed that methylene blue adsorption occurred within the MXene-PVA hydrogel network alongside MXene and PVA interactions [34].

**Figure 1:**
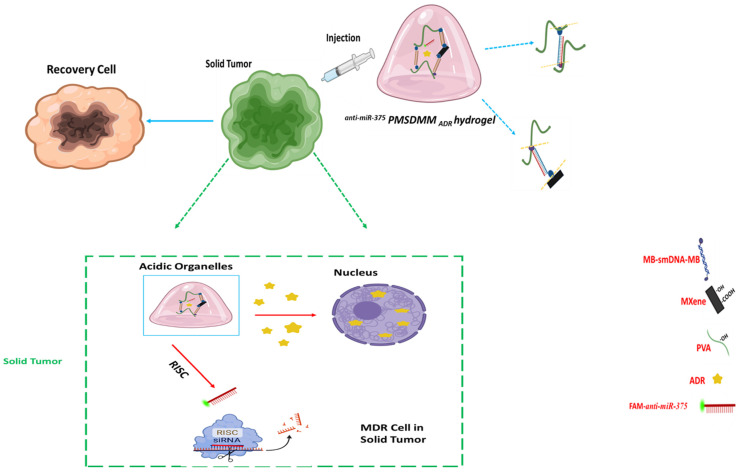
Schematic of the process of association of the pH-Sensitive ^anti-miR-375^ PMSDMM _ADR_ for delivery to solid tumors.

In this study, we used smDNA (double-stranded DNA) attached to MB to design a bio-inspired pH-sensitive hydrogel. At first, we created a PMSDMM hydrogel with a good tensile strength and swelling ratio and an MB-sm-DNA-MB biological crosslinking agent. We observed that MB can adsorb with PVA and also MXene, forming a unique platform for Adriamycin and miR-375 drug delivery. Here, the efficacy of the ADR-loaded PMSDMM-hydrogel (PMSDMM_ADR_), anti-miR-375-loaded PMSDMM-hydrogel (^anti-miR-375^ PMSDMM), and anti-miR-375-ADR co-loaded PMSDMM (^anti-miR-375^ PMSDMM _ADR_) was systematically investigated on MCF-7 breast cancer cells and ADR resistant MCF-7 (MCF-7/ADR) cells. The mechanism of overcoming MDR was also preliminarily investigated by testing the synergic affection of anti-miR-375 and ADR chemotherapy inhibition through study of the pH-sensitive mechanism. The current study designed an injectable pH-responsive ^anti-miR-375^ PMSDMM _ADR_ hydrogel, which we employed to enable the in situ application of coatings on solid tumors, to address vascular leaks and inadequate lymphatic drainage, and to rectify deficiencies following tumor removal. The pH-sensitive connections of methylene blue within these hydrogels facilitated drug release, specifically in the acidic environment that is characteristic of tumors. This method could be utilized to locally retain and regulate the release of medications aimed at suppressing tumor cell growth (Figure 1).

Differences between the values are represented as follows: * *p* ˂ 0.05, ** *p* ˂ 0.01 and *** *p* ˂ 0.001.

## 2. Materials and Methods

### 2.1. Materials

Lithium fluoride (LiF), Ti_3_AlC_2_ in powder form (200 mesh), Tris Buffer, Poly(vinyl alcohol), borax (sodium tetraborate decahydrate with a purity exceeding 99.5%, Na_2_B_4_O_7_·10H_2_O, Molecular weight = 381.37 g/mol), PVA with a molecular weight of between 26,300 and a degree of hydrolysis of 86.5 to 89%, MTT bromide, dimethyl sulfoxide (DMSO), a penicillin/streptomycin solution, sucrose, indomethacin, and sodium salt of deoxyribonucleic acid (smDNA) were sourced from Sigma (Sigma-Aldrich, St. Louis, MO, USA). Adriamycin (ADR) was prepared using materials from Calbiochem (Calbiochem, Shanghai, China). RPMI-1640 medium, along with fetal bovine serum (FBS) and 0.25% trypsin/EDTA, were obtained from Gibco (Gibco-BRL, Gaithersburg, MD, USA). Opti-MEM was acquired from Invitrogen (Invitrogen, Carlsbad, CA, USA). Small interfering RNA for miR-375 (anti-miR-375) and FAM-labeled anti-miR-375 were provided by Pishgam Biotech Co. in Mashhad, Iran. The primers required for real-time PCR were synthesized by Cinaclone Co. in Tehran, Iran. All compounds had a purity of over 95%, as determined by HPLC analysis.

### 2.2. Fabrication and Characterization of Ti_3_C_2_Tx Nanosheets

Ti_3_C_2_Tx MXene nanosheets were produced using a modified etching procedure with hydrofluoric acid (HF) [35,36]. In summary, 2.00 g of Ti_3_AlC_2_ powder was combined with 2.00 g of LiF in a 20 mL solution of hydrochloric acid (9 M). To eliminate oxygen, nitrogen gas was introduced and the mixture was sealed and placed in an oven at 200 °C for 24 h. Afterward, the resulting suspension was collected and washed again. The Ti_3_C_2_ product was isolated through centrifugal washing. Finally, the supernatant underwent freeze-drying to yield MXene nanosheets (Ti_3_C_2_), which were then stored at room temperature for subsequent transmission electron microscopy (TEM) analysis.

### 2.3. Synthesis of PMSDMM _ADR_, ^anti-miR-375^ PMSDMM, ^anti-miR-375^PMSDMM_ADR_ Hydrogels and PDMM Hydrogel as a Control Hydrogel

First, 0.8 g of PVA (10% *w*/*v*) was dissolved in 8 mL of distilled water, with continuous stirring at 95 °C until it was completely dissolved. Next, a solution containing 10 wt% deoxyribonucleic acid sodium salt (smDNA) in 4.0 mM NaBr was prepared and added to the PVA solution, maintaining stirring at 30 °C. ADR (5 mg/mL, dissolved in DMSO) was then introduced into this suspension, and the mixture was sonicated at room temperature for 30 min. To eliminate any unbound ADR molecules, the solution underwent centrifugation and filtration using 50 kDa MWCO amplicon filters, followed by washing with water until no discernible color remained in the filter solution. A separate 0.4 g portion of the prepared solution (10% *w*/*v*) containing a specific amount of MXene was dissolved in 8 mL of distilled water and stirred continuously at 95 °C until the PVA was fully dissolved. The resulting product was analyzed using UV-visible absorption spectroscopy (UV-vis) on a SHIMADZU UV2450 spectrometer (SHIMADZU, Kyoto, Japan). The concentration of the ADR loading was quantified using High-Performance Liquid Chromatography (HPLC) (LC-20A, SHIMADZU, Kyoto, Japan), equipped with an Agilent Eclipse XDB-C18 Reverse Phase Column (Santa Clara, CA, USA). Specifically, sample solution injection was conducted via a 20 mL sample loop. The mobile phase employed consisted of a mixture of acetonitrile and KH_2_PO_4_ (0.02 mol/L) in a volumetric ratio of 25:75 (*v*/*v*), with a flow rate set at 1.0 mL/min. The column was maintained at a constant temperature of 25 °C, and an ultraviolet detector (SPD-20A, SHIMADZU, Japan) operating at a wavelength of 254 nm, was used to monitor the column effluent. ^AntimiR-375^PMSDMM was prepared by gently mixing a predetermined quantity of RNA solution with the PMSDMM hydrogel solution for 30 min under ambient conditions. Similarly, ^anti-miR-375^PMSDMM _ADR_ was formulated by adding the desired amount of RNA solution into PMSDMM_ADR_ and mixing for half an hour at room temperature by sequential loading. The quantity of anti-miR-375 solution loaded onto PMSDMM was maintained equivalent to that loaded onto PMSDMM_ADR_. To evaluate the antimiR-375 loading efficiency on the PMSDMM_ADR_ complex, a gel retardation assay was conducted. Briefly, a fixed amount of anti-miR-375 was pipetted into varying volumes of PMSDMM_ADR_, ensuring optimal distribution. The resulting hydrogel complex was allowed to stand at room temperature for 30 min before further use. Subsequently, the complex was combined with RNA loading buffer (Takara, Dalian, China) and subjected to electrophoresis on a Urea PAGE gel composed of 20% polyacrylamide gel electrophoresis (PAGE) and 7M urea in 1× TBE buffer. The electrophoresis was carried out at 110 mV for 30 min. A gel documentation system (ChemiDoc XRS, Bio-Rad, Hercules, CA, USA) was utilized to analyze the gel, enabling assessment of the antimiR-375 complexation efficiency. For comparative analysis, the PDMM hydrogel was synthesized by following previously established protocols for the fabrication of Mxene-PVA hydrogels incorporating MB-ss DNA as a biologically functional cross-linker [34]. To this end, 0.6 g PVA (10% *w*/*v*) was heated at 90 °C for about 4 h, while 6 mg/mL of ss-MB-DNA was dissolved in 16 mL distilled water and stirred continuously at 30 °C until the DNA was completely dissolved without denaturation. Subsequently, a certain amount of MXene was added to the solution and stirred for 5 h. The hydrogel was stored in a desiccator to prevent moisture adsorption. This comparison was intended to help us to improve the affinity of these loading of drugs during the formation of ^anti-miR-375^PMSDMM _ADR_.

### 2.4. Characterization of ^anti-miR-375^ PMSDMM _ADR_ Hydrogel

To assess the mechanical properties of the ^antimiR-375^ PMSDMM _ADR_ hydrogels, samples were prepared using a dumbbell cutter (Analyzer Texture XT2i, Godalming, UK) with a thickness of 1 mm for tensile testing. The tensile tests were conducted at a speed of 10 mm/min to derive the stress–strain (σ) vs. strain curve (ΔL/L_0_), where σ was calculated based on the original cross-sectional area of the undeformed gel, and ΔL and L_0_ represent the deformation of the gel and its initial length, respectively. Thermal characterization (TGA) of pristine ^antimiR-375^ PMSDMM _ADR_ hydrogel nanocomposites was performed using a model SDTQ600 device (New York, NY, USA). The analysis involved a heating rate of 10 °C/min under a nitrogen atmosphere with a flow rate of 100 mL/min.

A UV-visible spectroscopy analysis of the ^antimiR-375^ PMSDMM _ADR_ hydrogels (10 mg dissolved in 1 mL distilled water) was carried out using a UV spectrometer (LABINDIA-3092, Thane, India). Additionally, the morphology of the hydrogels was examined through SEM (MERATESCAN, New York, NY, USA). To prepare the sample for SEM analysis, 10 mg/mL of the antimiR-375 PMSDMM ADR hydrogel powder was dispersed in deionized water (DD water) and stirred continuously. The resulting dispersion was placed onto a 400-gauge copper grid and air-dried for 5 min. The silver particle transmission electron microscope (TEM) image was captured using a Tecnai T-12 microscope operating at 80 kV (FEI, Cambridge, UK). The dimensions of the hydrogel pores were analyzed with ImageJ software (including its distribution Fiji, Version 1.53t). To evaluate the swelling behavior, a sample of the synthesized hydrogels was immersed in 100 mL of distilled water adjusted to specific pH levels. Four pH values were selected for this study: an acidic level of 2.0, a pH close to the pKa of acrylic acid (4.8), a neutral value of 7.4, and an alkaline level of 10.0. At predetermined time intervals, the samples were weighed repeatedly until a stable weight was reached for each. The percentage swelling ratio was determined using a specific equation, whereby Q represents the water absorption capacity (g of water per g of absorbent), Mt indicates the sample weight at time t (g), and M0 corresponds to the initial sample weight (g). Additionally, portions of the synthesized samples were immersed in 100 mL of distilled water at the designated pH levels for further analysis of swelling characteristics. Similar pH values were chosen—2.0, 4.8, 7.4, and 10.0—to maintain consistency. The samples were weighed at regular intervals and monitored until stabilization of their weights. The swelling ratio was computed using the corresponding equation:

(1) $$Q=\frac{M_{t}-M_{0}}{M_{0}}\times 100$$

where Q, *M_t_*, and *M*_0_ are the percentage of water absorption (g_water_/g_absorbent_), sample weight at time t (g), and initial sample weight (g), respectively.

### 2.5. Drug Loading/Release

The nanocomposite hydrogels to load drugs were prepared using the technique described above. The difference was that in loading anti-miR-375 and ADR into the ^anti-miR-375^ PMSDMM _ADR_ hydrogel with 5 mL of the ADR solution, additional steps we performed, as outlined previously. Our samples were poured into the dispersion of antimiR-375 and ^antimiR-375^ PMSDMM _ADR_ hydrogels during PMSDMM loading into ^antimiR-375^ PMSDMM _ADR_ hydrogels. UV-Vis spectrophotometry (with maxima at 275 nm and 425 nm), along with the calibration curve, were utilized to quantify the levels of anti-miR-375 and ADR in the corresponding media. To explore the drug release mechanisms, samples containing the drugs were individually immersed in two different media: neutral (pH = 7.4) and acidic (pH = 5.5). Additionally, a quantity of 0.05 g of hydrogel loaded with both drugs was submerged in 50 mL of these solutions. A rotating shaker set at 90 rpm was employed to facilitate the investigation of drug release at a temperature of 30 °C. Additionally, a quantity of 0.05 g of hydrogel loaded with both drugs was submerged in 50 mL of these solutions. A rotating shaker set at 90 rpm was employed to facilitate the investigation of drug release at a temperature of 30 °C. In the next step, 10 mg of each PMSDMM _ADR_ and ^antimiR-375^ PMSDMM (containing different amounts of anti-miR-375 and ADR) was dissolved in 1 mL of ethanol. All tubes were then shaken vigorously for 3 min, followed by 15 min of ultrasonication for complete disruption of the nanocarriers. The concentration of curcumin was measured by UV/Vis a spectrophotometer (Biochrom WPA Biowave II, Cambridge, UK) at 425 nm. The amount of curcumin was assayed based on the absorption relative to a calibration curve. Ultimately, assessments were made for the entrapment efficiencies (EEs) and DLC:

EE % = Weight of anti-miR-375 and ADR into CS MCM 41

Weight of feeding anti-miR-375 and ADR × 100

DLC % = Weight of anti-miR-375 and ADR into CS MCM 41

Weight total hydrogel × 100

### 2.6. Analysis of Cell Culture and Co-Delivery of ADR and Anti-miR-375 by PMSDMM into Cancer Cells

The ADR-resistant MCF-7/ADR breast cancer cell line, along with its parental MCF-7 cell line, were obtained from Royan Co. (Tehran, Iran). Both cell lines were cultivated in RPMI-1640 medium supplemented with 10% fetal bovine serum (FBS) and 1% penicillin/streptomycin at 37 °C in a humidified environment containing 5% CO_2_. Subculturing of the cells was performed routinely using trypsin/EDTA digestion once they had reached 80–90% confluence. To sustain the multidrug resistance (MDR) phenotype, 1000 ng/mL ADR was added to the medium; this supplementation was discontinued two weeks prior to experimental use. Dual-color flow cytometry was employed for the characterization of PMSDMM co-delivering ADR and anti-miR-375. MCF-7/ADR cells (1.6 × 10^5^ cells per well) were seeded in six-well plates until they reached approximately 70% confluence. FAM-anti-miR-375 PMSDMM ADR was prepared in the same manner as anti-miR-375 PMSDMM ADR. Other formulations, including PMSDMM, PMSDMM ADR, FAM-anti-miR-375 PMSDMM, and ^miR-375^ PMSDMM _ADR_, were added to the cells and incubated for 4 h at 37 °C. Following incubation, the cells were washed three times with PBS, trypsinized, collected, and resuspended in 500 μL PBS. The samples were analyzed using a FACS caliber flow cytometer (New York, NY, USA), with FL1 band-pass emission used to detect the green fluorescence in the FAM and FL2 band-pass emissions and the red fluorescence from ADR. Cells treated solely with the PMSDMM hydrogel served as the control group.

### 2.7. In Vitro Cytotoxicity Detection in MCF-7/ADR and MCF-7 Cells

Cells were seeded in 96-well plates at a density of 16,104 cells per well and subsequently subjected to treatments with ADR, ^antimiR-375^ PMSDMM, PMSDMM_ADR_, or a combination of ^antimiR-375^ PMSDMM _ADR_. Untreated cells served as controls for this experimental setup. The final concentrations utilized for antimiR-375, PMSDMM, and ADR were optimized at 0.25 mM, 1 mg/mL, and 1.75 mg/mL, respectively. Notably, the initial investigation indicated that PMSDMM did not exhibit significant cytotoxic effects at concentrations below 0.1 mg/mL. Following a 24-h treatment period, the medium was replaced with fresh growth media. Subsequently, an MTT assay was conducted by adding MTT reagent at a concentration of 5 mg/mL to each well, followed by an incubation period of 4 h. The culture medium was then removed, and 150 µL of DMSO was added to solubilize the formazan crystals. The plate was agitated for 20 s to ensure uniform dissolution, and absorbance readings were promptly recorded at 570 nm using an ELX800 absorbance microplate reader (Bio-Tek EPOCH, Winooski, VT, USA).

### 2.8. Expression Assay of miR-375 and ABCB1 in Vitro Assay

MCF-7 and MCF-7/ADR cells (16,105 cells per pore) were seeded into a six-well culture plate and allowed to grow until reaching approximately 70% confluence. The cells were then incubated for 24 h in a fresh medium containing either negative control RNA (ncRNA) or PMSDMMs loaded with ncRNA (termed ncRNA PMSDMMs) or ^antimiR-375^ PMSDMMs. The concentrations of PMSDMM and RNA in the medium were maintained at 1 mg/mL and 0.25 mM, respectively. Subsequently, the mature miR-375 levels within the cells were quantified using quantitative real-time PCR (qRT-PCR). Total RNA was extracted from the cultured cells using Trizol reagents (Invitrogen, Carlsbad, CA, USA). The quantification of mature miR-375 expression was performed with the SYBR Green miRNA assay, and the data were normalized using the 2^−ΔCt^ method, with human U6 serving as an endogenous control. Specifically, 1 µg of total RNA was reverse-transcribed into complementary DNA (cDNA) using AMV reverse transcriptase (TaKaRa, Dalian, China) and looped antisense primers. The reverse transcription procedure involved sequential incubation steps: 16 °C for 15 min, 42 °C for 60 min, and a final denaturation at 85 °C for 5 min to generate the miRNA cDNA. Real-time PCR analysis was conducted using an Applied Biosystems 7500 platform in accordance with standardized protocols. Each PCR amplification utilized 1 µL of synthesized cDNA in a reaction initiated by a five-minute pre-incubation at 95 °C, followed by 40 cycles of denaturation at 95 °C for 15 s and annealing/extension at 60 °C for one minute. The expression of ABCB1 was assessed using SYBR green-based detection, with normalization to human GAPDH expression via the 2^−ΔCt^ method. For reverse transcription of ABCB1, 2 µg of total RNA was utilized with AMV reverse transcriptase (TaKaRa), while real-time PCR expression analysis was performed using an Applied Biosystems 7500 system. All experiments were performed in triplicate to ensure the reproducibility and reliability of the data. Further technical details regarding protocols or instrumentation can be provided upon request.

### 2.9. Injection Mechanism in MCF-7/ADR Cells by ^antimiR-375^ PMSDMM _ADR_

To assess cellular uptake and drug retention, MCF-7/ADR cells were seeded into six-well plates at an initial density of 16,105 cells per well and allowed to grow until reaching approximately 70% confluence. At this stage, cells were treated with Adriamycin (ADR), PMSDMM _ADR_, and ^anti-miR-375^ PMSDMM _ADR_ for 24 h. Following the treatment period, cells were meticulously washed three times with ice-cold phosphate-buffered saline (PBS) to remove any residual substances. Subsequently, cells were lysed using 100 µL of Milli-Q water and homogenized with an Omni Sonic Ruptor 250 homogenizer (Omni, Kennesaw, GA, USA). A 20-µL aliquot of the resulting lysate homogenate was then prepared for high-performance liquid chromatography (HPLC) analysis. The intracellular concentration of ADR was quantified via a standard calibration curve and normalized to the protein content determined through a Pierce protein assay kit. To investigate the endocytic mechanisms underlying the uptake of ^anti-miR-375^ PMSDMM _ADR_, inhibition studies were performed. MCF-7/ADR cells were pretreated with either 0.4 M sucrose or 100 mM indomethacin at a low temperature (4 °C) prior to exposure to ^anti-miR-375^ PMSDMM _ADR_. The intracellular ADR uptake efficiency was subsequently evaluated by comparing the normalized ADR concentrations within lysates obtained from inhibitor-treated and non-treated groups relative to untreated controls. This experimental approach provided critical insights into the pathways influencing ADR delivery and retention in resistant cell lines.

### 2.10. Statistical Analysis

All experiments were conducted in three distinct stages, with the results presented as an average of ±SE. Statistical significance was assessed using the Student’s *t*-test.

## 3. Results and Discussion

### 3.1. MXene, PMSDMM_ADR_, ^anti-miR-375^ PMSDMM, and ^anti-miR-375^ PMSDMM _ADR_ Fabrication and Characterization

**Figure 2:**
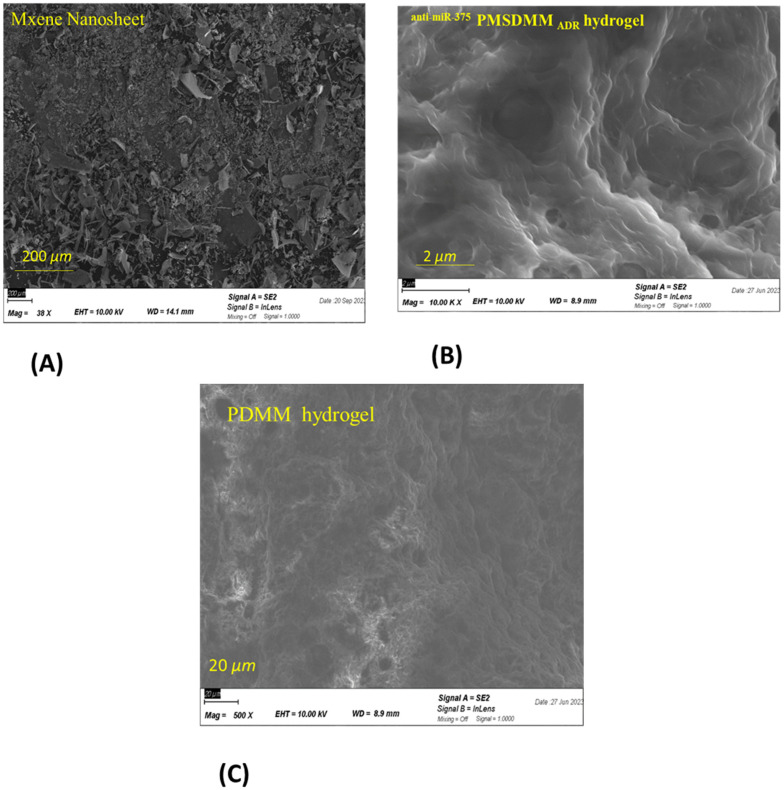
SEM Morphological properties of (**A**) nanosheets of MXene; (**B**) ^anti-miR-375^ PMSDMM _ADR_ hydrogels synthesized with MB-smDNA-MB cross linker; and (**C**) PDMM hydrogel synthesized by MB-DNA.

**Figure 3:**
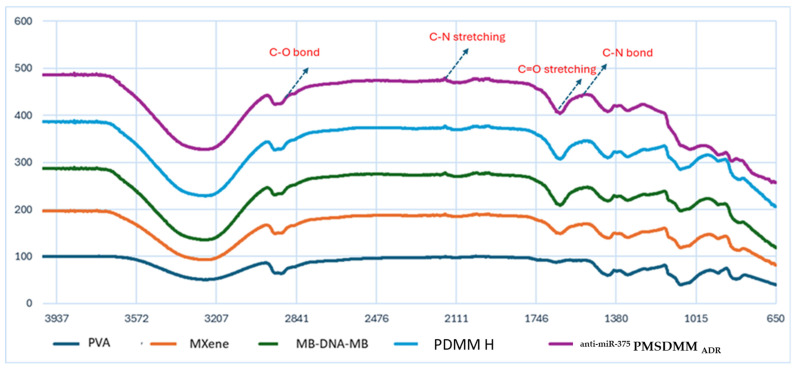
FT-IR spectra of the ^anti-miR-375^ PMSDMM _ADR_ hydrogel compared to the PVA, MXene, MB-DNA-MB, and PDMM hydrogels.

**Figure 4:**
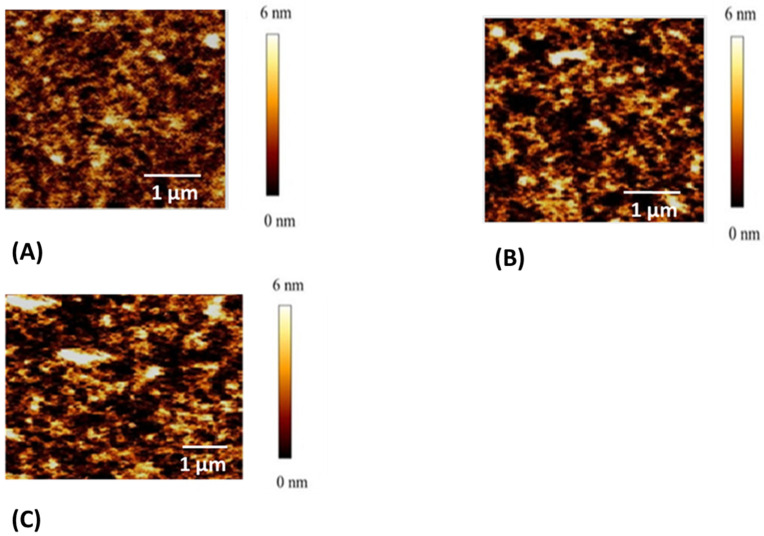
(**A**). AFM images of PDMM as a control (**B**,**C**) AFM images of ^anti-miR-375^ PMSDMM and ^anti-miR-375^ PMSDMM _ADR_.

Initially, the synthesized MXene nanosheets were derived from the MXene multilayer through the selective extraction of the aluminum layer using a LiF and HCl exfoliation technique (Figure 2A). Following this, upon introducing ADR and antimiR-375 molecules, hydrogels were created by forming inclusion complexes with multiple physical cross linking by the formation of a network of PVA-PVA chains along with MXene-PVA and smDNA, which acted as biological cross-linkers among PVA-PVA chains and PVA-MXene. Figure 2B,C display SEM images of the freeze-dried ^anti-miR-375^ PMSDMM _ADR_ hydrogel alongside PDMM hydrogel, which served as a control sample. In a previous study, we confirmed the creation of a multifunctional network within a DNA-bio-inspired hydrogel as a wearable skin sensor using MB-ssDNA-SH as a single strand of DNA (biological crosslinking agent) between PVA-MXene [34]. In that study, ^anti-miR-375^ PMSDMM _ADR_ formed during the loading of ADR and antimiR-375 molecules. This compound presented a significantly porous structure with double-stranded DNA (a more resistant DNA structure), which also acted as biological crosslinking agent with the ends of MB. This led to the formation of multiple networks of physical cross-linking bonds, yielding a solution that was suitable for injection. Many studies have demonstrated the formation of hydrogels composed of MXene/PVA using borax as a chemical agent [37,38,39]. Figure 3 shows the FTIR spectra of the PMSDMM hydrogel with MB-smDNA-MB compared to a PDMM hydrogel. The peak at 3445 cm^−1^ showed the presence of an OH expansion group (hydroxyl group), which indicated the presence of polyvinyl alcohol, MXene, and smDNA. FTIR peaks at 2225 cm^−1^ and 1600 cm^−1^ were observed sequentially, revealing C-N stretching and C=O stretching groups, respectively; this was attributed to the adsorption of MB on MXene and PVA. Wang et al. demonstrated the adsorption of MB on MXene using electrostatic forces [40]. Ahmed et al. described the adsorption relationship of MB in a pH sensitive starch hydrogel [41]. We also observed C-O, C–N, and N–H bonds at 2850 cm^−1^, 1600 cm^−1^, 1465 cm^−1^, respectively, in MB-smDNA-MB, indicating adsorption by PVA, Mxene, and Mxene sides, which may have indicated the presence of Mxene-Mxene and PVA-PVA, as well as Mxene-PVA. Our PMSDMM hydrogel showed peaks at 750 cm^−1^, 800 cm^−1^, and 2850 cm^−1^ due to the C-C stretching vibrations of the PVA-PVA network that formed in the borax hydrogel (Figure 3). A typical porous structure was observed in the obtained hydrogel; this is indispensable in drug delivery applications, as it allows drug diffusion to occur. The size of the final ^anti-miR-375^PMSDMM_ADR_ carrier was about 450 ± 645 nm (Figure 4C), as determined by AFM; see Figure 4A,B for comparisons with those of ^anti-miR-375^ PMSDMM and PMSDMM. A possible reason for this observation is that the ADR and antimiR-375 drug had been successfully loaded into the porous PMSDMM hydrogels before drying. In previous research on A431D cells (a cell line frequently utilized in cancer studies), a giant unilamellar vesicle (GUV) exhibiting a zeta potential of 31 mV demonstrated a nearly 100-fold enhancement in cellular attraction compared to GUV, with a zeta potential of +2 mV. In contrast, GUVs possessing a zeta potential of +28 mV have been shown to be 50 times more attractive than those with a zeta potential of +2 mV [42]. Therefore, the size of the ^anti-miR-375^PMSDMM_ADR_ was larger than the PMSDMM_ADR_ and the ^anti-miR-375^ PMSDMM. Since the morphology of the prepared PMSDMM loaded with ADR and antimiR-375 showed that the ^anti-miR-375^PMSDMM_ADR_ scales were less than 100 nm, the prepared hydrogels met the size requirements for efficient cancer therapy applications. Consequently, PMSDMM concentrations of 0.5 mg/mL were selected for subsequent studies.

The swelling properties of the ^anti-miR-375^ PMSDMM _ADR_ composite are illustrated in Figure S1. The hydrogel’s swelling ratio was monitored over a period of 30 min, after which it stabilized. This swelling behavior was attributed to the hydrophilic nature of the PVA present in the hydrogel matrix, which allowed for water molecule absorption from the local environment. These findings align with a study conducted by Hernandez and colleagues [43]. Additionally, Roko et al. demonstrated that multi-component hydrogels designed for methylene blue incorporation exhibited significant performance as a bacterial cellulose-based material, with their effects on swelling, adsorption capacity, and thermodynamic characteristics being quantifiably explicable [44]. The thermal stability of hydrogels combining borax and functional monomers such as Mxene and PVA and MB-smDNA-MB was investigated by TGA analysis (Figure S2). The TGA technique was performed by heating the samples under a nitrogen atmosphere in a range of temperatures, i.e., 100–600 °C. About 60 wt% of the PVA/smDNA/ADR was lost from 100 to 600 °C. This was attributed to the decomposition of PVA and PVA, as elevated residue levels were observed following treatment at temperatures below 600 °C, i.e., approximately 23.0 wt%. The maximum degradation rate (Tmax) occurred at around 348 °C. The inclusion of anti-miR-375 notably affected the thermal stability of the PMSDMM carriers. Ultimately, it was evident that with the incorporation of MXene, ^anti-miR-375^ PMSDMM _ADR_ displayed a marked enhancement in thermal stability compared to PVA, with higher residues under 800 °C, reaching around 42.0 wt% and an increase in T*max* up to about 420 °C.

**Figure 5:**
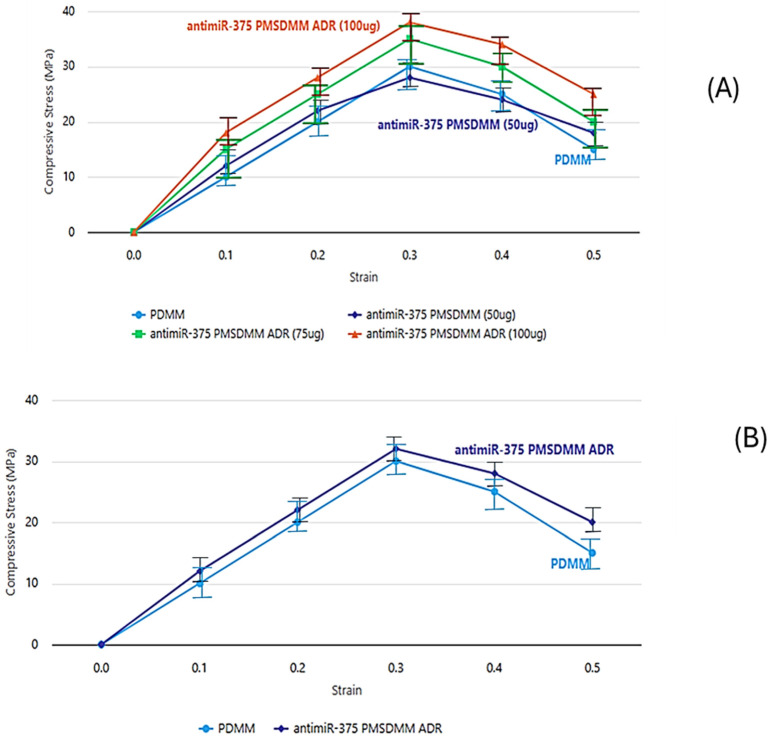
Compressive stress–strain curves of (**A**) ^antimiR-375^ PMSDMM_ADR_ (50 µg) gel, ^antimiR-375^ PMSDMM_ADR_ (75 µg), ^antimiR-375^ PMSDMM_ADR_ (100 µg) gel compared to the PDMM as a control. (**B**) Optimization for the compressive stress–strain curves of ^antimiR-375^ PMSDMM_ADR_ (60 µg) gel compared the PDMM as a control.

Compression stress–strain graphs are presented in Figure 5A,B. The compressive strength of the ^antimiR-375^ PMSDMM_ADR_ (100 µg) gel varied between 30 and 35 KPa, showing dependence on the ADR and antimiR-375 concentration. Furthermore, the compression modulus of ^antimiR-375^ PMSDMM_ADR_ (50 µg) gels decreased compared to that of PDMM gels. This was attributed to an unstable cross-link density resulting from the inclusion of ^AntimiR-375^ PMSDMM _ADR_ gels, with the highest observed stress being 28 kPa. These results may have been due to the abundance of hydroxyl groups in the antimiR-375 structure, which formed hydrogen bonds with PMSDMM. Generally, ^anti-miR-21^ PMSDMM _ADR_ indicated dependence on the ADR and antimiR-375 concentrations compared to the PDMM hydrogel. This may have been due to the increase in the cross-link density of the ADR and antimiR-375 structure during the formation of ^anti-miR-21^ PMSDMM _ADR_ as a consequence of the influence of MB-smDNA-MB and the addition of drugs. Here, we also optimized the control of ^antimiR-375^ PMSDMM_ADR_ (60 µg) gels compared to the PDMM gels. Our results showed stable cross linking in the form of MB and hydroxyl bonds in ^antimiR-375^ PMSDMM_ADR_ (60 µg) gels. In this study, we employed ultraviolet–visible (UV-vis) absorption spectroscopy to confirm the incorporation of ADR into PMSDMM. As illustrated in Figure S3A, ADR displayed a prominent absorption peak at 480 nm, whereas PMSDMM exhibited only a slight absorption peak at around 300 nm. The spectrum of PMSDMM_ADR_ distinctly revealed the characteristic absorption peak of ADR at 480 nm, which signified the successful formation of PMSDMM_ADR_. The quantity of ADR integrated into PMSDMM was measured using HPLC and was found to be 0.7 mg/mL. Furthermore, antimiR-375 was incorporated into PMSDMM_ADR_ through static interaction to produce antimiR-375 PMSDMM_ADR_, as outlined in the Methods Section 2. Finally, ^anti-miR-21^ PMSDMM _ADR_ had two strong absorption peaks between 180 nm and 800 nm: one at 450 nm, i.e., the typical peak of ADR, and the other at 260 nm, which indicated that anti-miR-375 was bound to PMSDMM _ADR_ (Figure S3A)_._ The ability of PMSDMM_ADR_ to form complexes with antimiR-375 was further studied using a gel retardation test. To that end, 500 pmol anti-miR-375 (100 mM, 5 mL) was added to a number of PMSDMM _ADR_ solutions. The results showed that significant interactions with anti-miR-375 occurred at a volume ratio of 0.8, and full complexation was observed at a volume ratio of 1.0 (Figure S3B). Thus, in all subsequent experiments, a volume ratio of 1.0 was selected.

### 3.2. Drug Loading and MB Adsorption Kinetics Through the Synthesis of pH-Sensitive ^anti-miR-375^ PMSDMM _ADR_

**Figure 6:**
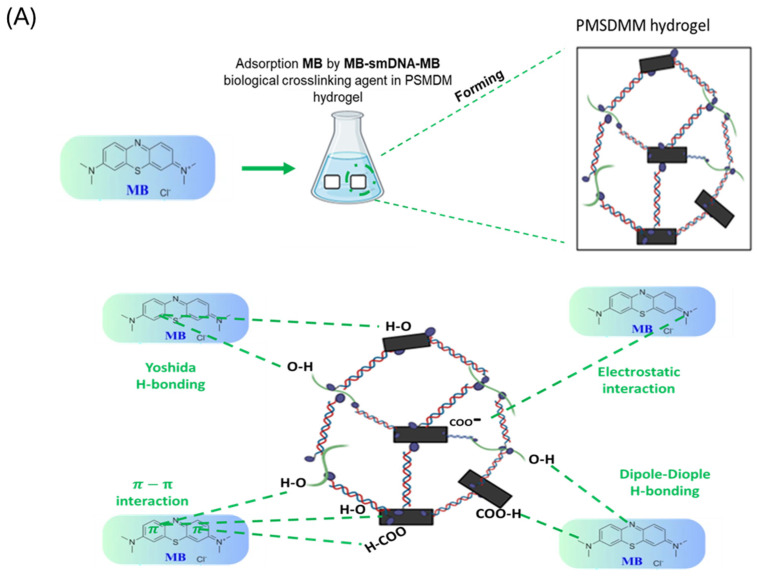

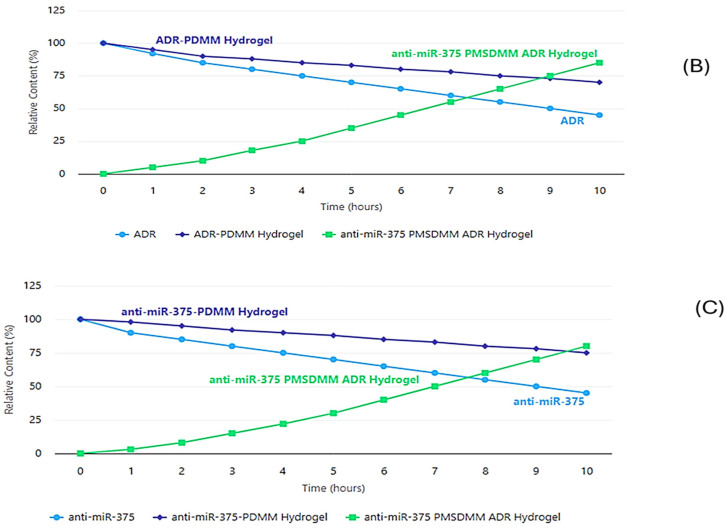
(**A**) Schematic image of the adsorption capability of MB for the ^antimiR-375^ PMSDMM _ADR_ hydrogel. (**B**,**C**) Stability of ADR and antimiR-375 during the formation of ^antimiR-375^ PMSDMM _ADR_ based on concentration.

Different amounts of anti-miR-375 and ADR were sequentially loaded into 10 mg PMSDMM in dispersion. The soluble ^antimiR-375^ PMSDMM_ADR_ complexes were filtered via a 0.22-lm filter (to remove insoluble curcumin). The ^antimiR-375^ PMSDMM_ADR_ complexes were freeze-dried to obtain solid complexes. EE and DLC (Figure S4) were measured at 88.1 ± 4.76% and 8.81 ± 0.47%, respectively, for ratios of 10% (ADR: MSDMM *w*/*w*) and 5% (anti-miR-375: MSDMM *w*/*w*), as quantitatively calculated using some equations. MB is broadly utilized as a textile dye, but it poses a risk to human health and safety [45]. In this study, we used it as a novel pH-sensitive carrier for the transformation of two drugs. In a previous study, we presented MB-ssDNA-OH as a biological crosslinking agent for the formation of a network in a Mxene-PVA hydrogel. We used single stranded DNA with the ends of MB and hydroxyl groups [34]. We demonstrated that MB can absorb PVA and Mxene. In that study, we used synthetic double-stranded DNA (sm-DNA) with MB at the ends to design a pH sensitive hydrogel. The adsorption energy of MB by hydrogels was found to be influenced by many factors, such as concentration, time, and crosslinking agents [46]. The effect of pH on the adsorption of MB-smDNA-MB was therefore investigated. The adsorption capacity of MB-smDNA-MB in a PMSDMM hydrogel based on pH is shown in (Figure S4). Our tests revealed that the release of ADR, antimiR-375, and ^antimiR-375^ PMSDMM _ADR_ at pH = 5.5 was greater than that at pH = 7.4. These findings indicate that a rise in pH led to improved adsorption performance. At lower pH levels, most of the carboxylic anions present in Mxene were protonated, forming COOH groups. In the case of PVA, hydroxyl groups formed OH entities. This resulted in the elimination of electrostatic repulsion between COO− and O− anions while enhancing hydrogen bonding interactions among polymer chains. Ultimately, this caused a contraction of the structure, making it more difficult for MB atoms to penetrate [47]. Moreover, interactions among carboxylate ions led to competition between surplus dissolved H^+^ ions and positively charged dye molecules, which limited adsorption at these lower pH levels. Adsorption at low pH (below the isoelectric point) was constrained to the hydrogen bonds formed between OH or COOH groups on polymer chains and amine groups on MB [48]. At elevated pH values, both Mxene’s carboxyl and PVA’s hydroxyl groups were deprotonated into negatively charged carboxylate and hydroxylate ions. The resulting electrostatic repulsions among these negatively charged sites within the hydrogel prompted structural expansion and enhanced electrostatic attractions with positively charged components in MB [49]. Our investigation revealed that various factors contributed to the adsorption capacity; these included the dye solution concentration, the effects of cross-linking agents, and pore sizes within the synthesized hydrogel (Figure 6A). The curve in Figure 6B,C shows that the concentrations of ADR in the ADR solution and PDMM hydrogel were 75–80% after 10 h. The antimiR-375 encapsulated into PMSDMM also remained stable (Figure 6C). These results may have been due to the fact that our PMSDMM hydrogel was formed by a process of encapsulation of antimiR-375 and also ADR. We therefore suggest parallel repetition of the experiment to ensure data rigor.

On one hand, the main reason for the low bioavailability of ADR is its low water solubility. On the other hand, these data indicate that the release of ^antimiR-375^ PMSDMM _ADR_ gels was strongly correlated with pH, which is an important point for the application of ^antimiR-375^PMSDMM _ADR_ in acid tumor microenvironments. Physical crosslinking mechanisms like hydrogen bonding, ionic interactions, or hydrophobic interactions can also be exploited in injectable hydrogels [50,51]. Our hydrogel with pH-sensitive bonds enabled the release of drugs into the acid tumor microenvironment via an injectable hydrogel. This platform could be used for the local immobilization and continuous release of drugs to inhibit the proliferation of tumor cells.

### 3.3. Co-Delivery of ADR and Anti-miR-375 by Injection of PMSDMM into Cancer Cells

**Figure 7:**
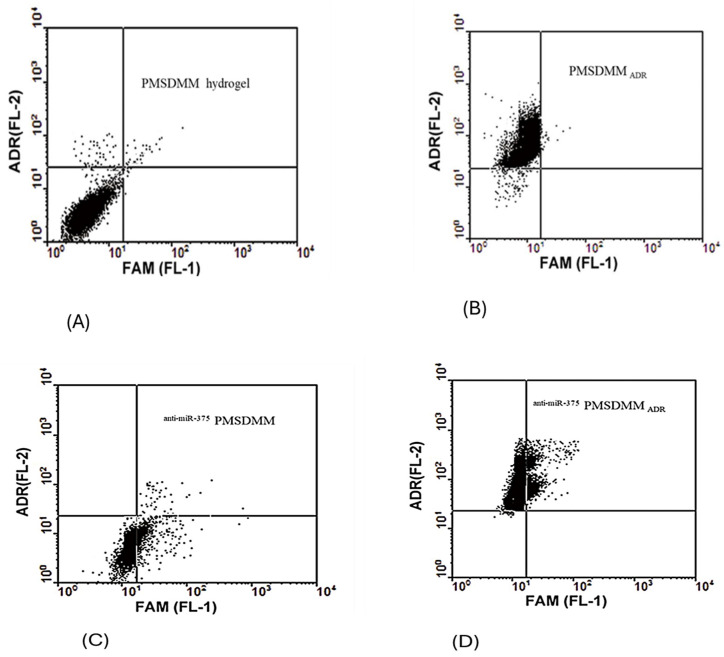
Characterization by flow cytometry of ^anti-miR-21^ PMSDMM _ADR_. MCF-7/ADR cells were incubated with PMSDMM _ADR_, ^FAM-anti-miR-21^PMSDMM, and ^FAM-anti-miR-21^ PMSDMM _ADR_. (**A**–**D**) After incubation at 37 °C for 4 h, the cells were collected for flow cytometry analysis. Cells treated with blank PMSDMM were utilized as a control group.

One of the most important criteria in combined cancer therapies is the ability to evaluate the efficacy of carriers in terms of drug delivery to cells [52]. Here, we evaluated the co-delivery of anti-miR-375 and ADR by PMSDMM to MCF-7/ADR cells. Our flow cytometry results showed, based on red fluorescence, that ADR had been successfully delivered to cancer cells (Figure 7A,B). Meanwhile, green fluorescence in cells indicated that PMSDMM could also deliver FAM-labelled anti-miR-375 to MCF-7/ADR cells (Figure 7C). Finally, the combined colors of cells were detected after ^FAM-antimiR-375^ PMSDMM _ADR_, indicating that PMSDMM was able to deliver ADR and antimiR-375 simultaneously to MCF-7/ADR cells (Figure 7D). Our findings indicate that it is possible to administer both chemotherapeutics and gene therapy agents within the same cancer cells through the injection of PMSDMM. Numerous studies have documented intelligent methods for co-delivery, including a noteworthy case where P-glycoprotein siRNA and doxorubicin were successfully co-delivered to HeLa cells using quantum dots to counteract MDR [53,54]. PLGA nanoparticles were developed for the co-delivery of Paclitaxel and Stat3 siRNA to overcome resistance in lung cancer cells [55]. There are nonetheless numerous challenges associated with solid tumor microenvironments (TMEs), including regulatory T cells (Tregs), myeloid-derived suppressor cells (MDSCs), tumor-associated macrophages (TAMs), inhibitory cytokines such as IL-10 and TGF-β, hypoxic conditions, and obstacles to efficient drug delivery, like elevated intra-tumoral pressure [56]. Local interventions—including surgical procedures, radiotherapy, localized ablation, and regional drug administration—can improve the effectiveness of chimeric antigen receptor T cell therapy [57,58]. To advance local drug delivery methods, Li et al. demonstrated the use of an injectable hydrogel matrix (Gel@MSN/miR-21-5p) which allowed for the controlled release of microRNA-21 triggered by the surrounding acidic environment [59]. In our investigation, we observed that this co-delivery hydrogel system could achieve drug release from an ^anti-miR-375^ PMSDMM _ADR_ hydrogel based on pH levels—a crucial aspect for utilizing potential carriers like hydrogels within acidic tumor microenvironments.

### 3.4. In Vitro Cytotoxicity Analysis of MCF-7/ADR Cells with ^antimiR-375^ PMSDMM _ADR_

**Figure 8:**
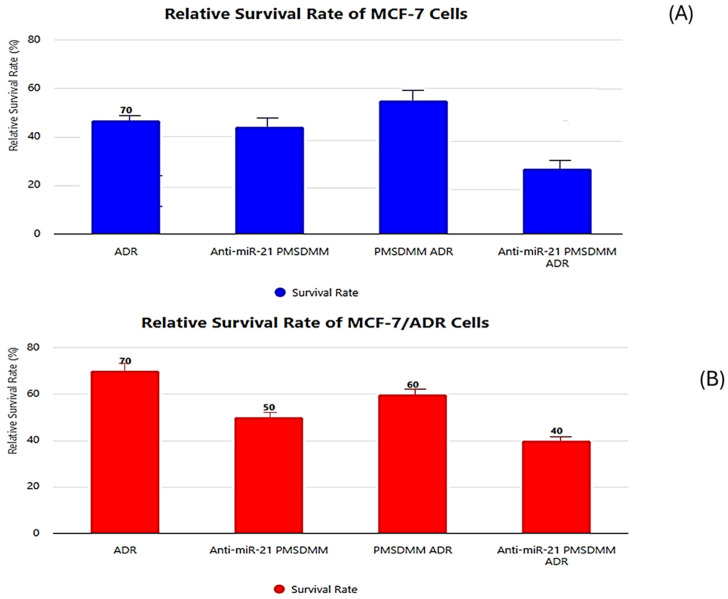
In vitro cellular cytotoxicity. Relative survival rate of MCF-7 cells (**A**) and MCF-7/ADR cells (**B**) after being treated with ADR, ^anti-miR-21^ PMSDMM, PMSDMM _ADR_, and ^anti-miR-21^ PMSDMM _ADR_ for 24 h. Control samples comprised untreated MCF-7 and MCF-7/ADR cells in each experiment. Data are means six SD for three separate experiments.

In this study, we utilized an MTT assay to evaluate the differential cytotoxicity of ADR, ^anti-miR-375^ PMSDMM, PMSDMM _ADR_, and anti-miR-375 PMSDMM ADR on MCF-7 cells (ADR-sensitive) and MCF-7/ADR cells (ADR-resistant). Culturing protocols with both drug treatments were conducted for 24 h. The data revealed that the cytotoxicity of free ADR was significantly lower in MCF-7/ADR cells compared to MCF-7 cells, attributed to drug resistance in MCF-7/ADR cells (Figure 8A). On the other hand, the results indicated that anti-miR-375 PMSDMM slightly reduced the survival rates in both MCF-7 and MCF-7/ADR cells. This reduction may suggest the action of miR-375 siRNA in inhibiting cancer cell proliferation. Interestingly, the survival rate of MCF-7 cells remained consistent at approximately 20% when treated with PMSDMM ADR and free ADR. Conversely, in MCF-7/ADR cells, the survival rate after treatment with PMSDMM ADR decreased significantly, i.e., to 62%. Notably, anti-miR-375 PMSDMM ADR further reduced the survival rate in MCF-7 cells from 40% to 20%, while in MCF-7/ADR cells, it decreased from 62% to 48% (Figure 8B). These findings demonstrate that anti-miR-375 PMSDMM ADR effectively counteracted drug resistance in MCF-7/ADR cells. Additionally, our data suggest that inhibiting specific miR-375 siRNA enhanced the cytotoxicity of chemotherapeutic agents like ADR, offering potential benefits for combating solid tumors.

The resistance of tumors to specific drug treatments may be impacted by the regulatory role of miRNAs. Research indicates that often, blocking certain miRNAs can enhance the cytotoxic effects of chemotherapy agents on cancer cells [60,61]. For instance, when miR-21 is inhibited alongside 5-fluorouracil treatment, a noticeable reduction in cell proliferation was observed in glioma and colorectal cancers. Additionally, hydrogels have been identified as innovative carriers that can stabilize the cytotoxic effects on solid tumors following injection therapy. Our findings align with those of Zhi et al., who demonstrated that functionalized graphene oxide could effectively deliver Adriamycin and anti-miR-21 to combat multidrug resistance [59].

### 3.5. Confirmation Analysis of ^antimiR-375^ PMSDMM _ADR_ to Show Resistance of MDR Activity

To confirm the enhancement of the anticancer activity of ADR by effective inhibition of miR-375 expression, we used a novel strategy of co-delivery of antimiR-375 and ADR by ^anti-miR-375^ PMSDMM _ADR_ to tackle the resistance of MDR cells to ADR_._ To detect the expression levels of miR-375 in MCF-7 and MCF-7/ADR, qRT-PCR tests were carried out after 24 h of treatment with ncRNA PMSDMM and ^anti-miR-375^ PMSDMM _ADR_. Our results confirmed that the overexpression of miR-375 in a MDR breast cancer line—by the relative expression of miR-375 in MCF-7/ADR—was shown to be higher than the corresponding level in the MCF-7 cell line (Figure S5A).

The injection of ^anti-miR-375^ PMSDMM _ADR_ significantly decreased the expression level of miR-375 in both MCF-7 and drug-resistant MCF-7/ADR cells, showing reductions of 55% and 40%, respectively, compared to the negative control. These findings confirmed that ^anti-miR-375^ PMSDMM _ADR_ effectively delivered anti-miR-375 to cancer cells and inhibited miR-375 expression. Post-treatment, the expression levels in MCF-7/ADR cells dropped by 40% and 55%, respectively, indicating that silencing miR-375 through ^anti-miR-375^ PMSDMM _ADR_ injections may reduce ABCB1 levels, thereby aiding in overcoming multidrug resistance (MDR), as shown in Figure S6A,B. Previous studies have showcased that employing short hairpin RNA expression vectors alongside magnetic Fe_3_O_4_ nanoparticles can effectively reverse multidrug resistance in leukemia cells by modulating the ADR and MDR1 pathways [62]. Further research in this area has led to the development of an innovative nanocomplex through the non-covalent adsorption of ADR and anti-miR-21 onto PPG, maintaining the structural integrity and functionality of both agents while supporting their controlled release within cells [63]. Additionally, functionalized graphene oxide demonstrated efficacy in co-delivering miR-21-targeted siRNA and ADR to cancer cells in vitro, providing substantial evidence for the utility of multifunctional nanoformulations in combating multidrug resistance [59]. Moreover, nanocarrier systems for ADR delivery have shown promise in mitigating toxicity and reducing adverse effects.

### 3.6. Evaluation of Drug Accumulation During the Injection Mechanism of ^anti-miR-375^ PMSDMM _ADR_ in MCF-7/ADR Cells

In situ-forming hydrogels are injectable as a solution; they then turn into a gel at the application site [15]. To confirm that the injection led to the in situ formation of ^anti-miR-375^ PMSDMM _ADR_ to enhance cytotoxicity, we tested the effects of accumulation of ^anti-miR-375^ PMSDMM _ADR_ in MCF-7and MCF-7/ADR cells. As shown in Figure S6, the accumulation of ADR in MCF-7/ADR cells, injected in the form of PMSDMM_ADR_, was higher than in cells treated with free ADR due to the efflux of P-gp drug being partially reduced by the introduction of PMSDMM. The injection of anti-miR-375 together with ADR in the form of ^anti-miR-375^ PMSDMM _ADR_ significantly increased the accumulation of ADR, i.e., to a level approximately 3.1 times higher than observed with the ADR solution. Our findings showed the distinct effect of injecting *^anti-miR-375^ PMSDMM _ADR_*. This could indicate the simultaneous formation of an *^anti-miR-375^ PMSDMM _ADR_* hydrogel loaded with two potent drugs, thereby enhancing the effect of ADR accumulation. Zhi et al. showed the accumulation of ADR to a level about 2.1-fold greater than that observed with ADR solution and 1.68-fold higher than that of PPG_ADR_ alone, indicating the ability of ^anti-miR-21^PPG_ADR_ to overcome MDR through the co-delivery of ^anti-miR-21^PPG_ADR_ [59]. Generally, these results are evidence of the ability of pH-sensitive *^anti-miR-375^ PMSDMM _ADR_* to overcome MDR, as opposed to simply counteracting the efflux of drug transporters on cell membranes.

## 4. Conclusions

In the realm of cancer treatment, one of the biggest challenges faced by clinicians is the phenomenon of multi-drug resistance (MDR) in solid tumors [12,64]. Resistance to traditional chemotherapy drugs significantly hinders the effectiveness of treatment, making it difficult to combat the progression of these aggressive cancers [65,66]. However, a promising new sustainable approach has emerged, i.e., the in situ coating of one or more drugs and miR in the form of hydrogels, offering a potential solution to this complex problem [67,68]. Rong et al. used as a co-delivery system comprising camptothecin and MiR-145 in the form of lipid nanoparticles for MRI-visible targeted therapy of hepatocellular carcinoma. The LA-CMGL-mediated co-delivery of miR-145 and CPT displayed a synergistic effect against HCC [69].

The use of hydrogels for drug delivery offers several key advantages. First and foremost, hydrogels can be engineered to exhibit specific properties, such as controlled release kinetics and the ability to target specific tissues [70]. This level of precision allows for the localized delivery of drugs directly to the tumor site, minimizing off-target effects and reducing systemic toxicity. Furthermore, hydrogels provide a stable and biocompatible platform for the co-delivery of multiple agents [71]. Wang et al. presented miR-222-engineered extracellular vesicles (TeEVs), tailored with cardiac-ischemia-targeting peptides (CTPs), as ischemic TeEV therapeutics. Those TeEVs were encapsulated within mechanical hydrogels to create injectable TeEV-loaded cardiac patches, enabling minimal invasiveness to attenuate IRI [72]. Solid tumors possess a series of biological barriers in the cellular microenvironment, necessitating the development of drug delivery methods based on advanced stimuli-responsive materials. In this study, the main goal was to apply a chemo-gene drug-delivery system with controlled drug release into solid tumors in order to predict treatment efficacy [73].

We showed that by incorporating both ADR and miR-375 within the hydrogel matrix of Mxene and smDNA, thereby forming a smart *^anti-miR-375^ PMSDMM _ADR_* platform, we could effectively target both the proliferating cancer cells and the mechanisms underlying MDR. This dual-targeting approach, i.e., Adriamycin and miR-375, showed the ability to bypass resistance mechanisms and improve the treatment’s therapeutic effectiveness using in situ-formed injectable hydrogels that responded to pH conditions. Additional research is needed to refine the formulation and assess both the safety and effectiveness of this co-delivery system in preclinical and clinical trials. Nevertheless, progress in nanotechnology and gene therapy offers significant hope for future cancer therapies, providing optimism for countless patients across the globe.

## Data Availability

All of data was in the main manuscript and Supplementary Materials. For a raw data, will be accessible from the corresponding authors.

## Supplementary Materials

The following supporting information can be downloaded at: https://www.mdpi.com/article/10.3390/pharmaceutics17070823/s1, Figure S1. Swelling behavior of ^anti-miR-375^ PMSDMM _ADR_ hydrogel composite compare to PDMM hydrogel as a control. Figure S2. Thermal stability of ^anti-miR-375^ PSMDM _ADR_ hydrogel composite relative to temperature melts of Mxene, PVA. Figure S3. Verification of the synthesis of ^antimiR-375^ PMSDMM_ADR_ hydrogel. (A) UV-Vis spectra of ADR, PVA-smDNA, PVA-smDNA-ADR and ^antimiR-375^ PMSDMM_ADR_ hydrogel in aqueous solution; (B) Electrophoretic mobility of anti-miR-375 with ^antimiR-375^ PMSDMM_ADR_ hydrogel at different volume ratios. Figure S4. Cumulative release of ADR and Antimir-375 in different solution and pH. Figure S5. In vitro expression of miR-375 relative to control hydrogel(^ncRNA^ PMSDMM) and ^anti-miR-21^ PMSDMM _ADR_. Real time PCR analysis of relative miR-375 expression (A) in MCF-7 and (B) MCF-7/ADR cells treated with ^ncRNA^ PMSDMM as a control and ^anti-miR-21^ PMSDMM _ADR_. ^ncRNA^ PMSDMM acted as a control for showing the expression of miR-375. Figure S6. ADR accumulation and uptake mechanism in MCF-7/ADR cells. ADR accumulation in MCF-7/ADR cells incubated with ADR, PMSDMM _ADR_ or anti-miR-21_._ PPGADR for 24 h. The ADR concentration was determined by HPLC and normalized to total cell protein. Data are shown as the mean 6 SD, n = 3. Data are means 6 SD for three separate experiments, ** *p* ˂ 0.01, *** *p* ˂ 0.001.
