# Supplementary material for: Engineered Sustainable Mxene-PVA Hydrogel as an Inspiring Co-Delivery Carrier for Targeting Solid Tumors
Source: Pharmaceutics. 2025 Jun 25;17(7):823. doi: 10.3390/pharmaceutics17070823 (PMC12300088; doi:10.3390/pharmaceutics17070823)
Supplement: Supplementary file 1 [file pharmaceutics-17-00823-s001.zip › pharmaceutics-3659860-supplementary.pdf]

Engineered Sustainable Mxene-PVA Hydrogel as an Inspiring Co-Delivery Carrier for Targeting Solid Tumors

Elham Ghazizadeh [1,2,*], Mahya Sadeghi [3], Hans-Peter Deigner [4] and Ali Neshastehriz [2,*]

[1] Department of Bioinspired Materials and Biosensor Technologies, Institute of Materials Science, Faculty of Engineering, Kiel University, 24143 Kiel, Germany

[2] Radiation Biology Research Center, Iran University of Medical Sciences (IUMS), Tehran 1416634793, Iran

[3] Department of Tissue Engineering, Faculty of Advanced Technologies, Tehran University of Medical Sciences, Tehran 14155-6559, Iran

[4] Institute of Precision Medicine, Furtwangen University, Jakob-Kienzle-Strasse 17, 78054 Villingen-Schwenningen, Germany

* Correspondence: elgh@tf.ac.ir (E.G.); neshastehriz@yahoo.com (A.N.)

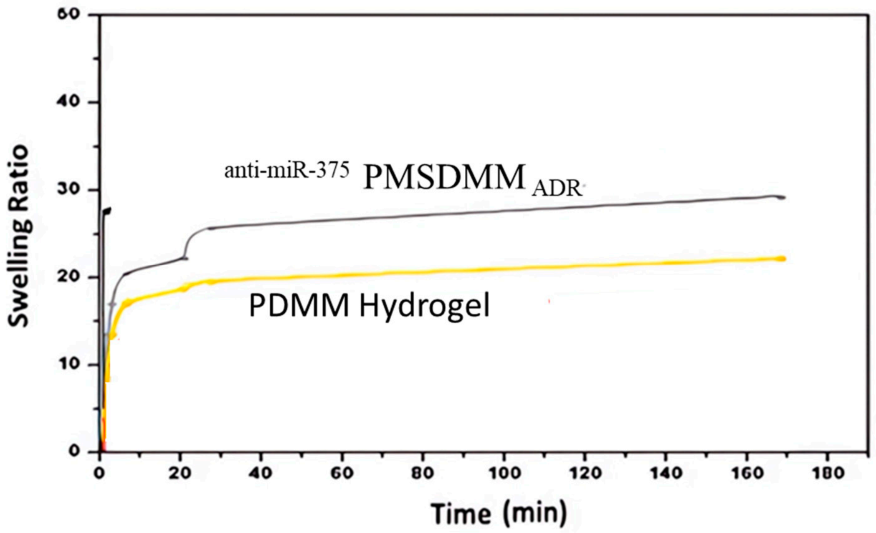

Figure S1. Swelling behavior of anti-miR-375 PMSDMM $_{ADR}$ hydrogel composite compare to PDMM hydrogel as a control.

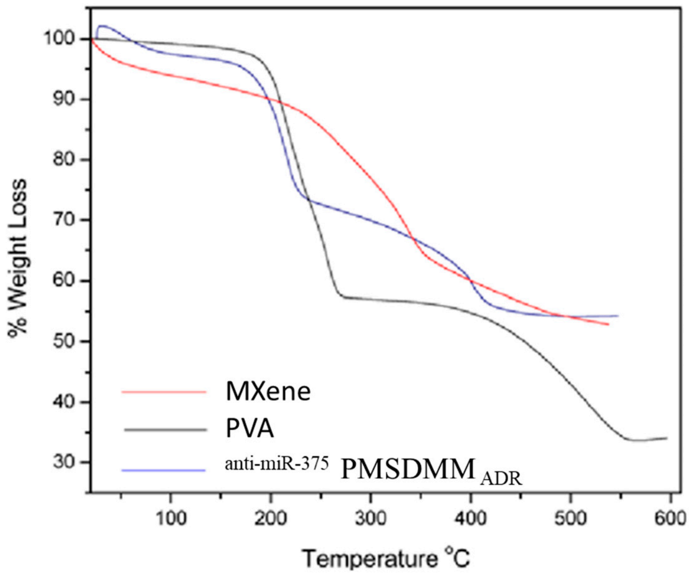

Figure S2. Thermal stability of anti-miR-375 PSMDM $_{ADR}$ hydrogel composite relative to temperature melts of Mxene, PVA.

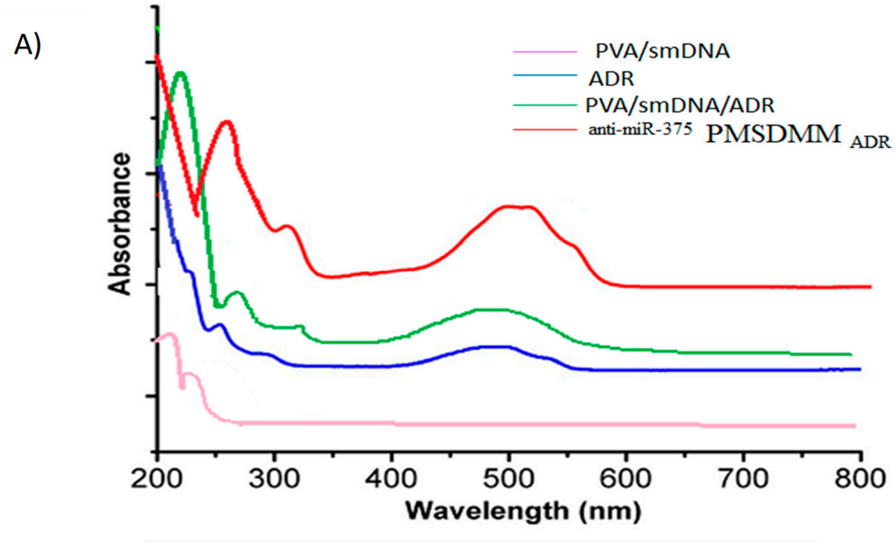

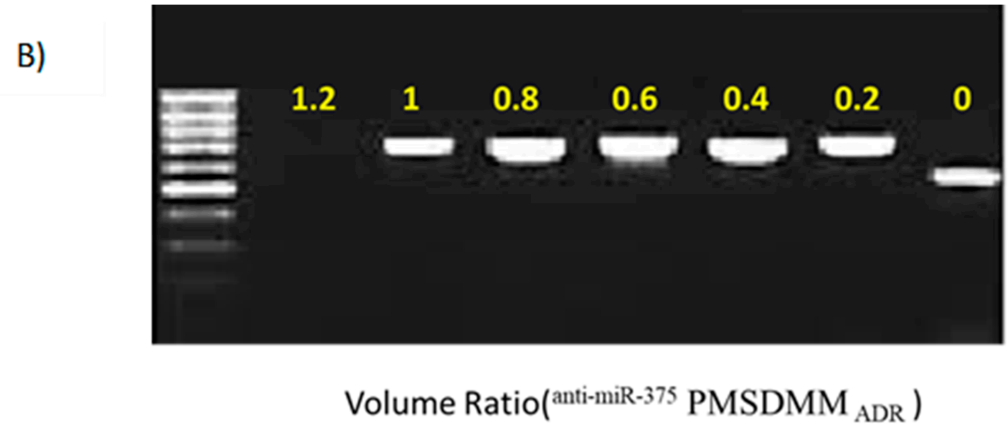

Figure S3. Verification of the synthesis of antimiR-375 $PMSDMM_{ADR}$ hydrogel. (A) UV-Vis spectra of ADR, PVA-smDNA, PVA-smDNA-ADR and antimiR-375 $PMSDMM_{ADR}$ hydrogel in aqueous solution; (B) Electrophoretic mobility of anti-miR-375 with antimiR-375 $PMSDMM_{ADR}$ hydrogel at different volume ratios.

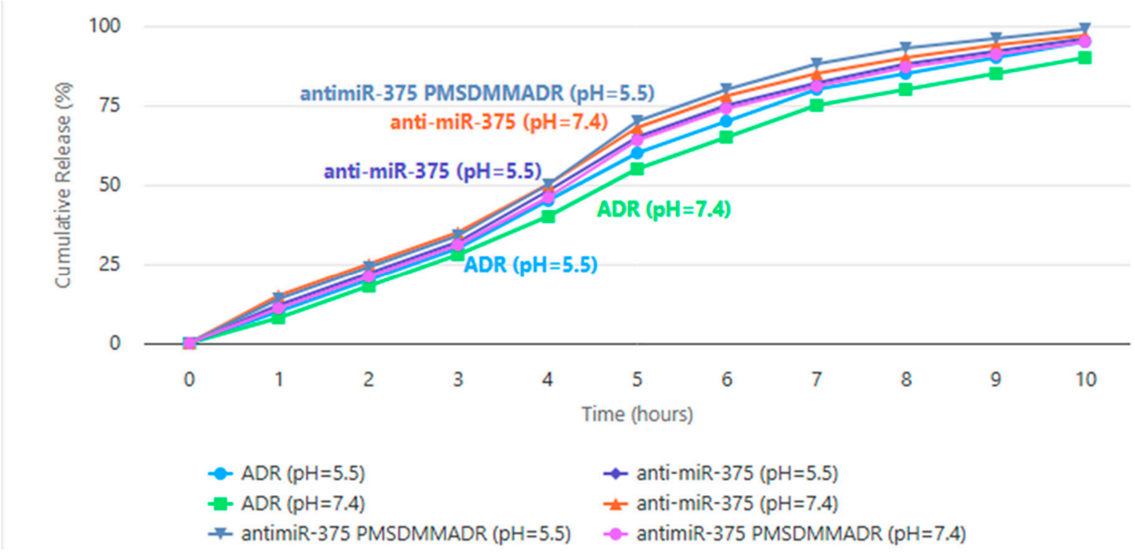

Figure S4.Cumulative release of ADR and Antimir-375 in different solution and pH

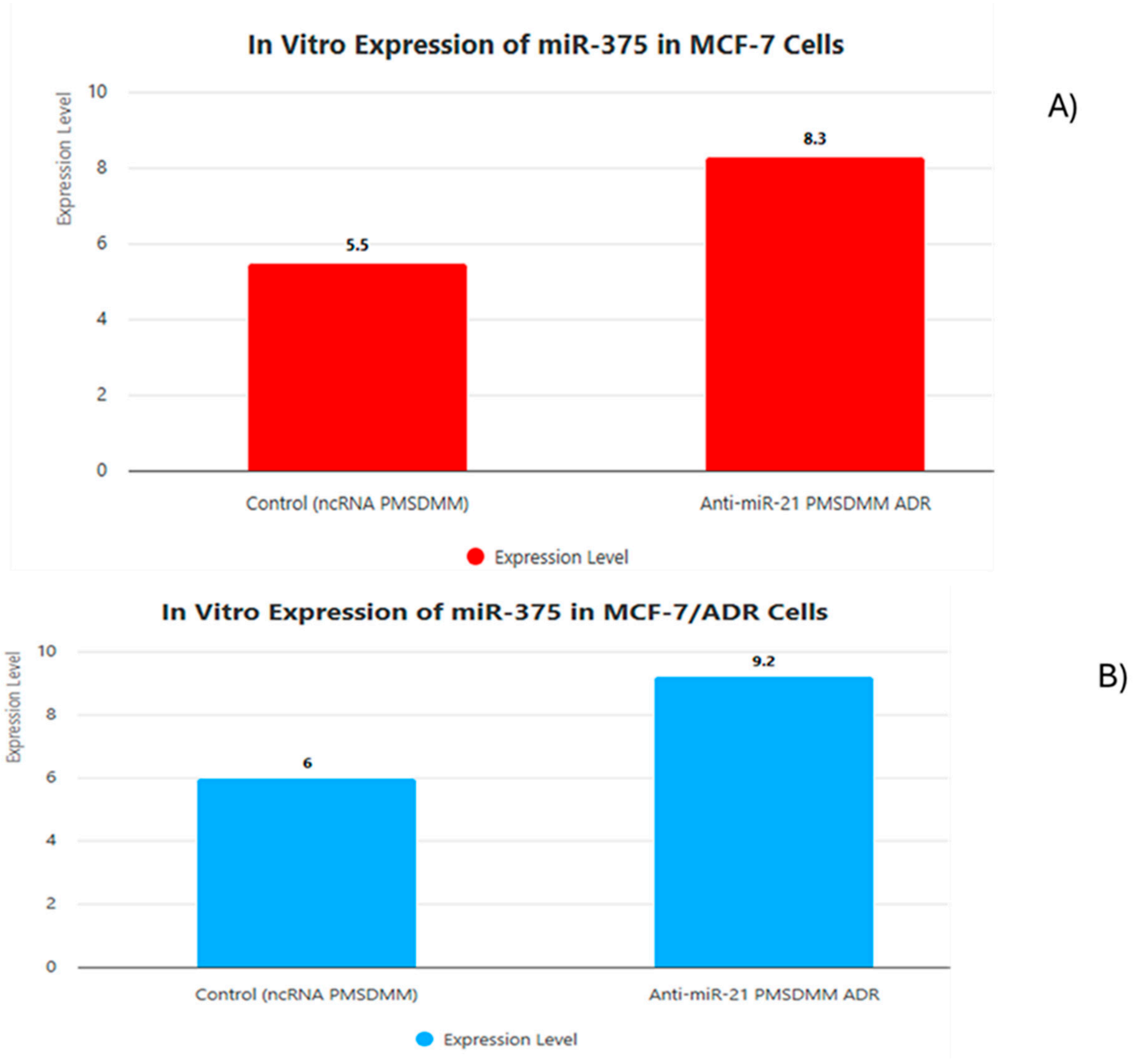

Figure S5. In vitro expression of miR-375 relative to control hydrogel(^{ncRNA} PMSDMM) and $^{anti\text{-}miR\text{-}21}$ PMSDMM $_{ADR}$. Real time PCR analysis of relative miR-375 expression (A) in MCF-7 and (B) MCF-7/ADR cells treated with ^{ncRNA} PMSDMM as a control and $^{anti\text{-}miR\text{-}21}$ PMSDMM $_{ADR}$. ^{ncRNA} PMSDMM acted as a control for showing the expression of miR-375.

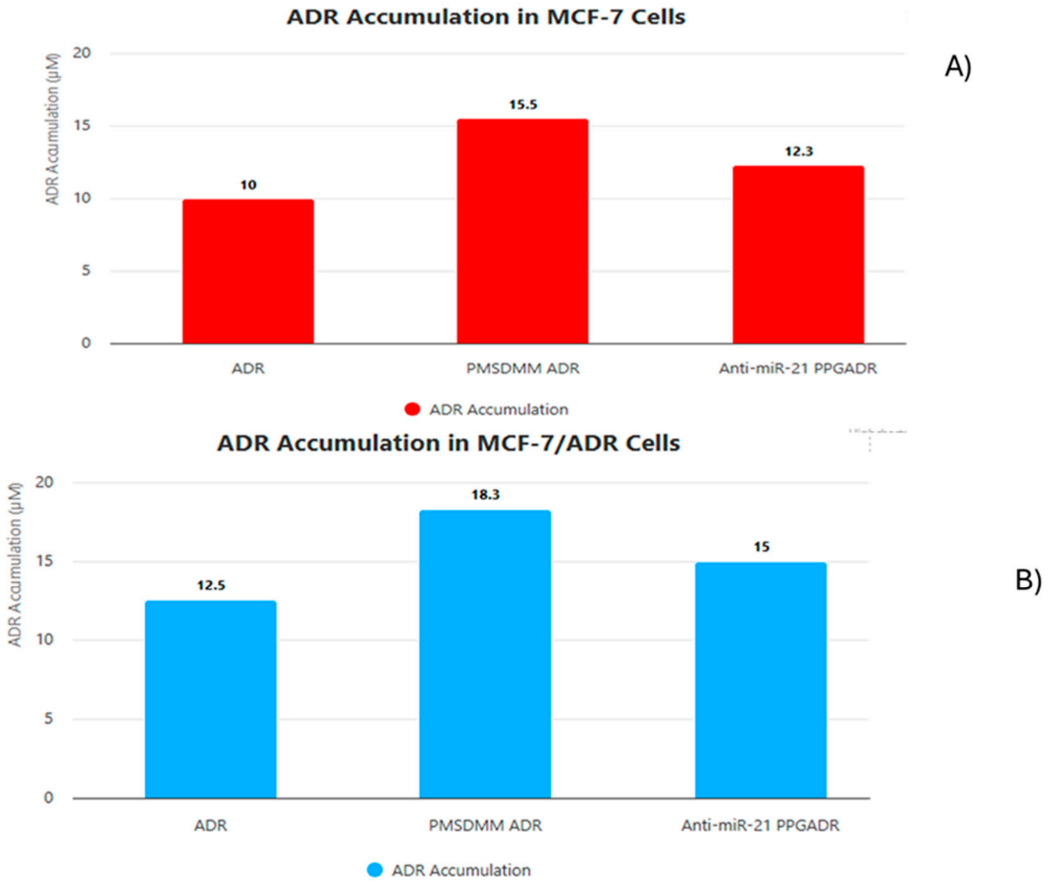

Figure S6. ADR accumulation and uptake mechanism in MCF-7/ADR cells. ADR accumulation in MCF-7/ADR cells incubated with ADR, PMSDMM $_{ADR}$ or anti-miR-21. PPGADR for 24 h. The ADR concentration was determined by HPLC and normalized to total cell protein. Data are shown as the mean 6 SD, n = 3. Data are means 6 SD for three separate experiments, **P<0.01, ***P<0.001.
